# Investigation into the role of H2-Ab1 in vascular remodeling in pulmonary arterial hypertension via Bioinformatics

**DOI:** 10.1186/s12890-024-03156-w

**Published:** 2024-07-15

**Authors:** Guowen Wang, Zhuoyan Wang

**Affiliations:** 1 Department of Respiratory Medicine, Affiliated Hospital of Shaoxing University, No. 999 South Zhongxing Road, Shaoxing, Zhejiang 312000 China; 2 Center for General Practice Medicine, General Practice and Health Management Center, Zhejiang Provincial People’s Hospital (Affiliated People’s Hospital), Hangzhou Medical College, No. 158 Shangtang Road, Hangzhou, Zhejiang 310014 China

**Keywords:** Pulmonary arterial hypertension, Gene expression omnibus, H2-Ab1, SU5416/hypoxia, Angiogenesis

## Abstract

**Background:**

Pulmonary arterial hypertension (PAH) is a progressive disease of vascular remodeling characterized by persistent pulmonary arterial pressure elevation, which can lead to right heart failure and premature death. Given the complex pathogenesis and poor prognosis of PAH, the identification and investigation of biomarkers become increasingly critical for advancing further understanding of the disease.

**Methods:**

PAH-related datasets, GSE49114, GSE180169 and GSE154959, were downloaded from the publicly available GEO database. By performing WGCNA on the GSE49114 dataset, a total of 906 PAH-related key module genes were screened out. By carrying out differential analysis on the GSE180169 dataset, a total of 576 differentially expressed genes were identified. Additionally, the GSE154959 single-cell sequencing dataset was also subjected to differential analysis, leading to the identification of 34 DEGs within endothelial cells. By taking intersection of the above three groups of DEGs, five PAH-related hub genes were screened out, namely Plvap, Cyp4b1, Foxf1, H2-Ab1, and H2-Eb1, among which H2-Ab1 was selected for subsequent experiments.

**Results:**

A SuHx mouse model was prepared using the SU5416/hypoxia method, and the successful construction of the model was evaluated through Hematoxylin-Eosin staining, hemodynamic detection, fulton index, and Western Blot (WB). The results of WB and qRT-PCR demonstrated a significant upregulation of H2-Ab1 expression in SuHx mice. Consistent with the results of bioinformatics analysis, a time-dependent increase was observed in H2-Ab1 expression in hypoxia-treated mouse pulmonary artery endothelial cells (PAECs). To investigate whether H2-Ab1 affects the development and progression of PAH, we knocked down H2-Ab1 expression in PAECs, and found that its knockdown inhibited the viability, adhesion, migration, and angiogenesis, while concurrently promoted the apoptosis of PAECs.

**Conclusion:**

H2-Ab1 could regulate the proliferation, apoptosis, adhesion, migration, and angiogenesis of PAECs.

**Supplementary Information:**

The online version contains supplementary material available at 10.1186/s12890-024-03156-w.

## Background

Pulmonary hypertension (PH) refers to a pathophysiological and clinical syndrome characterized by increased pulmonary artery pressure and pulmonary vascular resistance caused by a variety of heterogeneous diseases with different pathogenesis. It can progress to right heart failure and, in severe cases lead to death [1]. PH is clinically divided into five categories, pulmonary arterial hypertension (PAH) [2], PH due to left heart disease [3], PH due to hypoxia or pulmonary disease [4], chronic thromboembolic pulmonary hypertension (CTEPH) or other obstructive pulmonary disease [5], and unknown or multifactorial PH. PAH is a devastating and rare disease with complex pathogenesis and extremely poor prognosis. The pathogenesis of PAH involves pulmonary vasoconstriction, small vessel occlusion, thickening and large vascular obstruction. The presence of vascular lesions in PAH can lead to increase in pulmonary vascular resistance and right heart failure, often resulting in death if PAH patients left untreated [6]. At present, the clinical pharmacotherapy for PAH comprises prostacyclin pathway agonist, endothelin pathway antagonist, and phosphodiesterase-5 inhibitor [2]. Although the use of these drugs significantly improved patient survival rates, PAH remains a chronic progressive disease. Clinical practitioners always rely on their extensive clinical experience to determine appropriate treatment strategies, including how to use drugs clinically and the potential need for surgical interventions. By conducting this study, we expect to develop new disease biomarkers to monitor the development of PAH, thereby providing a scientific basis for the clinical assessment of the disease.

Endothelial cells (ECs) play roles in the progression and development of PAH. They affect vasoconstriction, inflammation, blood clotting, metabolism and oxidation/nitrification stress, as well as the pathophysiological processes of cell vitality, growth, and differentiation [7]. Dysfunction of ECs can aggravate vascular inflammation, leading to vascular remodeling and right ventricular (RV) failure [8]. Existing studies have found that the disorder of nitric oxide (NO) signaling pathways and reactive oxygen species (ROS) can also result in abnormal proliferation of pulmonary artery smooth muscle cells (PASMCs) and pulmonary artery endothelial cells (PAECs), which in turn leads to vascular remodeling, metabolic abnormalities, and DNA damage [9]. Moreover, ECs and smooth muscle cells can jointly mediate vascular remodeling and PH through signaling interactions [10–12].

In this study, we downloaded PH-related datasets from GEO, performed differential analysis and WGCNA, and screened out five hub genes: Plvap, Cyp4b1, Foxf1, H2-Ab1, and H2-Eb1. Based on their expression patterns and relevant literature review, H2-Ab1 was selected for subsequent verification. Our experiments confirmed a significant upregulation of H2-Ab1 in SuHx mice and hypoxia-treated mouse PAECs. To further verify the role of H2-Ab1, we knocked down H2-Ab1 in PAECs in vitro, and examined the proliferation, apoptosis, adhesion, and angiogenesis of PAECs. In summary, we aim to identify new disease biomarkers for PAH through bioinformatics analysis and experimental detection, thereby providing a scientific basis for improving the clinical assessment of PAH.

## Methods

### Data acquisition

We retrieved PH-related datasets, GSE49114, GSE180169 and GSE154959, from the publicly available database GEO (https://www.ncbi.nlm.nih.gov/geo/). All samples in these datasets were lung tissues. There were eight PH and eight control samples in the GSE49114 dataset, four PAH and five control samples in the GSE180169 dataset, and three PAH and three control samples in the GSE154959 dataset. Subsequently, we performed data quality control and filtering using the fastp software (version 0.20.1) with default parameters. Additionally, the hisat2 software (version 2.2.1) was used to compare data with mouse genome reference sequence (version GRCm38), and the “Rsubread” package (version 2.12.3) in R was used to calculate the count number and transcript per million (TPM) value of the gene.

### Differential analysis

Differential analysis was conducted on the GSE180169 dataset using the “edgeR” package (version 3.40.2). The “clusterProfiler” package (version 4.6.2) was applied for performing GO and KEGG enrichment analyses on the differentially expressed genes (DEGs).

### WGCNA

The “WGCNA” package was used for performing WGCNA on the GSE49114 dataset. We performed sample clustering on the TCGA dataset, removed the outlier samples, and then calculated the correlation of gene expression. Subsequently, we carried out sub-scale operations, set the soft threshold (i.e., the value of sub-scale) to 18 (R^2^ = 0.87), and built a scale-free network. The differential genes were constructed into an adjacency matrix with R^2^ = 0.87, and then transformed into a topological overlap matrix. A total of nine modules were identified.

### Single-cell analysis

The Seurat (version 4.3.0) and “tidyverse” package (version 2.0.0) were used for performing single-cell analysis on the GSE154959 dataset. Data were filtered based on the criteria of 200 < nFeature < 2000, percent.mt < 10 and nCount < 5000. Subsequently, the “harmony” package (version 0.1.1) was used for performing batch effect correction. Finally, cell annotation was carried out using the “SingleR” package (version 2.0.0), and the results were displayed by UMAP.

### Experimental animal

Healthy adult male C57BL/6 mice at 8 weeks of age, weighing 23–26 g, were purchased from Hangzhou Medical College Laboratory Animal Center. The mice were kept under constant temperature and humidity, and subjected to a 12 h alternating light–dark cycle. This animal experiment plan received approval from the experimental animal welfare ethics committee (Approval Number: ZJCJA-IACUC-20,010,260), in line with national laboratory animal welfare ethics rules. The mice were euthanized using CO_2_ asphyxia.

### Animal model preparation and grouping

Twelve experimental mice were equally and randomly divided into two groups: the control and the SuHx groups. Mice in the SuHx group underwent induction using the SU5416/hypoxia (SuHx) method, involving weekly subcutaneous injections of 20 mg/kg SU5416 for 3 weeks, with exposure to chronic hypoxia (10% oxygen). In contrast, mice in the control group received weekly subcutaneous injections of corresponding blank solvent and were raised in oxygen environment.

### Hematoxylin-eosin (HE) staining

We dewaxed the paraffin sections of the mouse lung tissue to water, stained them with hematoxylin and eosin (Beyotime, C0105M), dehydrated and sealed the slices, and photographed them to observe the density of the tumor tissue.

### Hemodynamic detection

The mice were anesthetized using Zoletil (50 mg/kg) and fixed on the operating table in the supine position. After disinfection, an incision was made in the median neck of the mouse to expose the trachea, which was then intubated and connected to an animal mechanical ventilator for assisted ventilation. Next, a chest incision was made to expose the heart, and a 20-gauge trocar was used for RV puncture. The trocar was connected to a pressure transducer through an extension catheter, and the pressure transducer was connected to an electrocardiogram monitor for the measurement of RV systolic pressure.

### Cardiac hypertrophy index

The hearts of the mice were isolated after euthanasia with CO_2_. Following the removal of the atria, auricle, and proximal connective tissue with scissors, the ventricles was thoroughly rinsed for several times in cold PBS to remove blood from the surface and chambers. Subsequently, we isolated the right ventricle and blotted it dry. We weighed the RV and left ventricular + septum (LV + S) separately, and then calculated the RV/ (LV + S) ratio.

### Western blot (WB)

Total protein was extracted from cells using a cell lysate containing 1% protease inhibitor, and the concentration of the extracted proteins was quantified using the BCA method. Subsequently, we carried out electrophoresis, followed by membrane transfer, antibody incubation, and colour development. H2-Ab1 (Biocompare, #MBS9143467), Bax (cell signaling technology1, #2772), and Bcl2 (cell signaling technology, #3498) were primary antibodies, and GAPDH (cell signaling technology, #5174) was selected as the internal reference gene.

### qRT-PCR

The TRIzol kit was used to extract total RNA from lung tissues or cells. cDNA was obtained using the PrimeScript RT kit (TaKaRa, Japan), and then diluted with RNase-free water. Quantitative PCR was performed using the SYBR Green PCR kit. The primer sequences used in this experiment are shown in Table 1.

**Table 1:** Primer sequences

Primer	Sequences
H2-Ab1-F	5′-TCACTCCAGGCTACAGAACTTTGC-3′
H2-Ab1-R	5′-TCTAAGGCACAGGTAATGGCAGTC-3′
GAPDH-F	5′-TGAAGCAGGCATCTGAGGG-3′
GAPDH-R	5′-CGAAGGTGGAA GAGTGGGAG-3′

### Cell model construction and grouping

The PAECs (Procell, #CP-M267) were subjected to hypoxic conditions (1% O_2_, 5% CO_2_, 94% N_2_) to mimic PAH in vitro, and PAECs under normoxia conditions (21% O_2_, 5% CO_2_, 74% N_2_) served as the control. We silenced H2-Ab1 in PAECs in one group, and concurrently transfected PAECs with empty plasmid (sh-NC) in another group as a control. Subsequently, the successfully transfected PAECs were treated with hypoxia for 72 h, and they were designated as the hypoxia + sh-H2-Ab1 group and the hypoxia + sh-NC group, respectively.

### CCK8

Cells in the logarithmic phase of growth were used to prepare a cell suspension, which was cultured for 24 h and then treated with CCK-8 reagent (solarbio, #CA1210) for an additional 2 h. The absorbance at 450 nm of the cell suspension was detected using an enzymoleter.

### Cell adhesion assay

Cells in the logarithmic phase of growth were used to prepare a cell suspension, which was then cultured for 3 h, and then incubated with 100 µL of Calcein UltraGreen AM (AAT Bioquest, #23,010) for 30 min.Subsequently, the cells were rinsed with PBS and finally detected with fluorescent enzyme labeling to evaluate cell adhesion.

### Wound healing assay

After culturing a monolayer of adherent cells in the dish, we removed cells in the central portion by marking the central region of cell growth. After 48 h of continuous culture, we observed whether the peripheral cells migrated to the central scratched area under a microscope to assess cell migration.

### Tube formation assay

Pre-cooled Matrigel was added to a 24-well plate, followed by addition of 500 µL of cell suspension into each well of the plate. Then, the cells were routinely cultured in a cell incubator for 4 h, and the tube formation ability of the cells was observed.

### Statistical analysis

We conducted data analysis and created bar graphs using the GraphPad prism 9. Measurement data were expressed as mean ± standard deviation (x ± s), and *t*-test was used to assess sample differences between two groups. *p* < 0.05 was considered statistically significant.

## Results

### Differential analysis

**Fig. 1:**
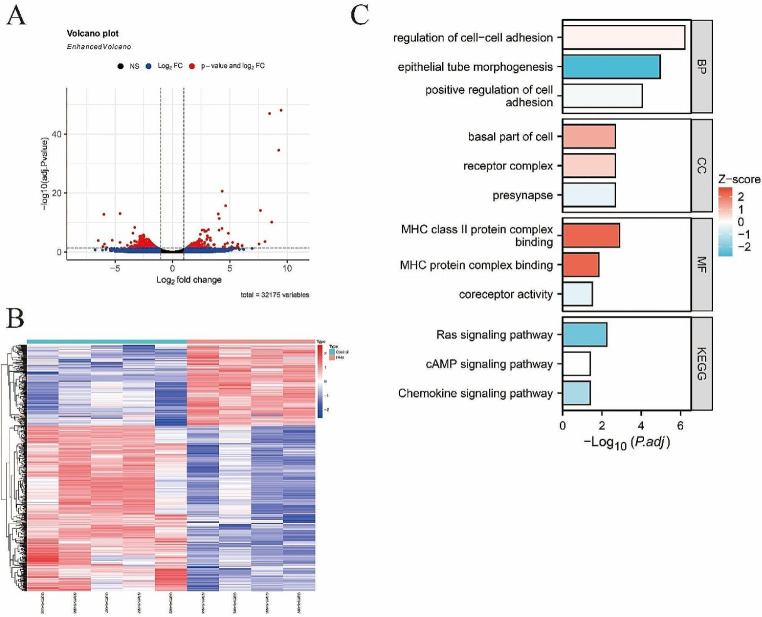
Differential analysis. (**A**). Volcano plot; (**B**). Heatmap; (**C**). Bar chart of functional analysis. *Note* BP: Biological Process; CC: Cellular Component; MF: Molecular Function

Differential analysis was performed on the GSE180169 dataset, based on the criteria of |log_2_FC|>1 & adj.Pvalue < 0.05 for standard screening, after which a total of 576 DEGs were screened out, including 388 down-regulated and 188 up-regulated ones (Fig. 1A-B). Subsequently, GO and KEGG analysis were performed on the DEGs, revealing enrichment of the genes predominantly in the regulation of cell-cell adhesion, positive regulation of cell adhesion, Ras pathway, cAMP pathway, and chemokine signaling pathway (Fig. 1C).

### WGCNA

**Fig. 2:**
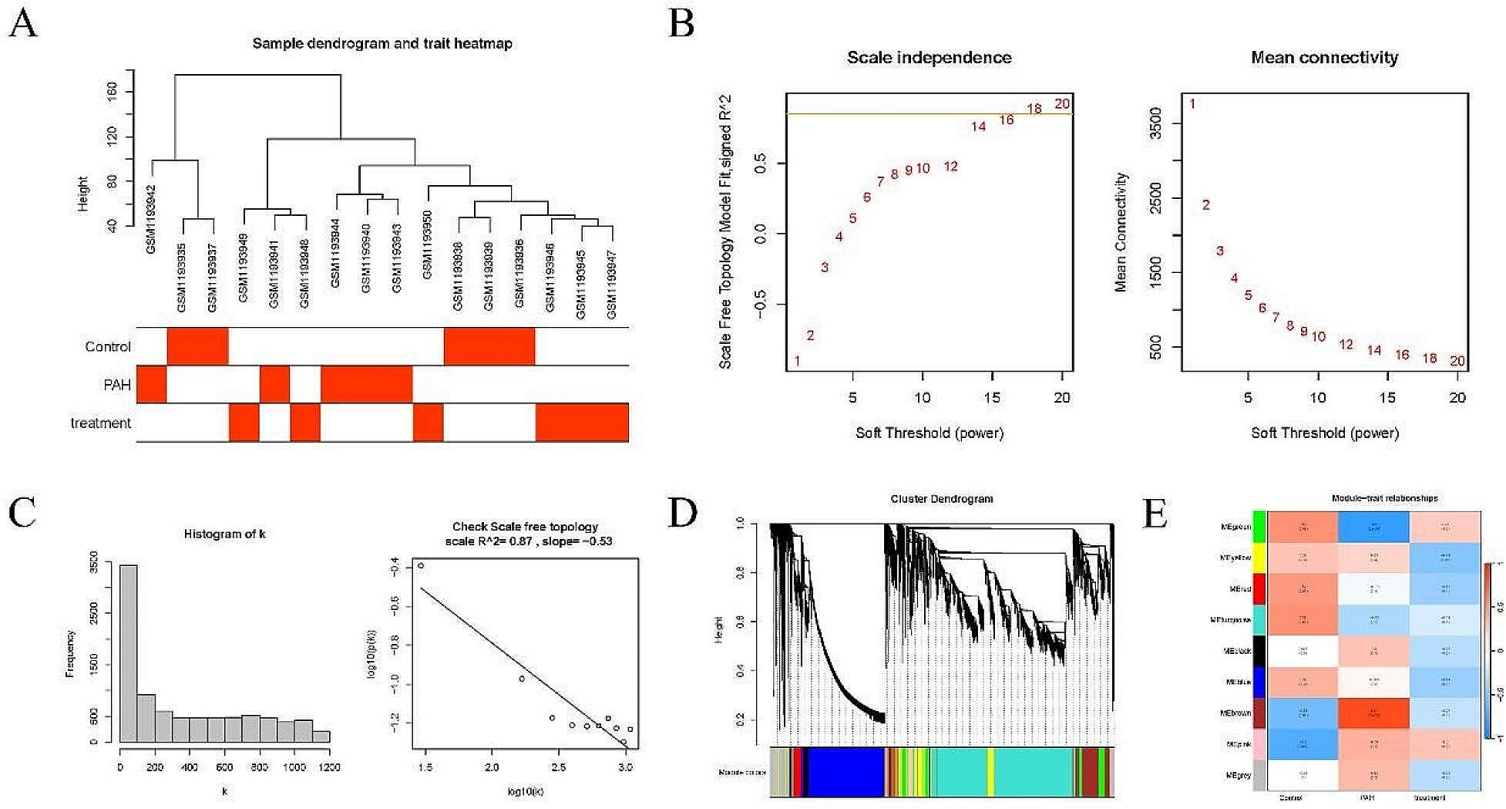
WGCNA. (**A**). Heatmap of sample characteristics; (**B**). The average connectivity; (**C**). Scale-free topology; (**D**). Cluster phylogenetic tree of modules; (**E**). Heatmap of correlation between each group and each module

**Fig. 3:**
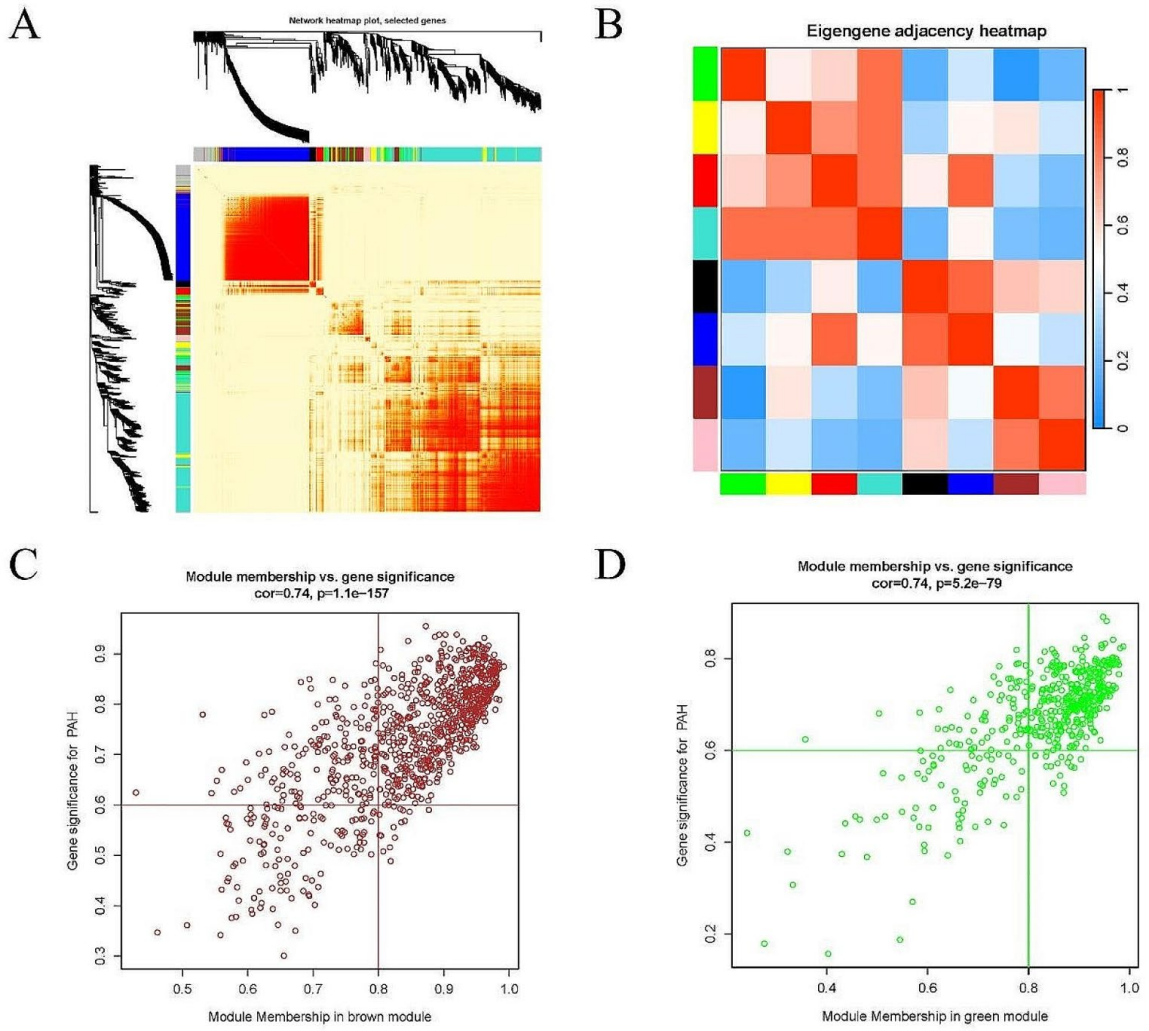
Correlation analysis. (**A**). Correlation heatmap; (**B**). Heatmap of module correlation; (**C**). Scatter plot of GS vs. MM for the brown module; (**D**). Scatter plot of GS vs. MM for the green module

**Fig. 4:**
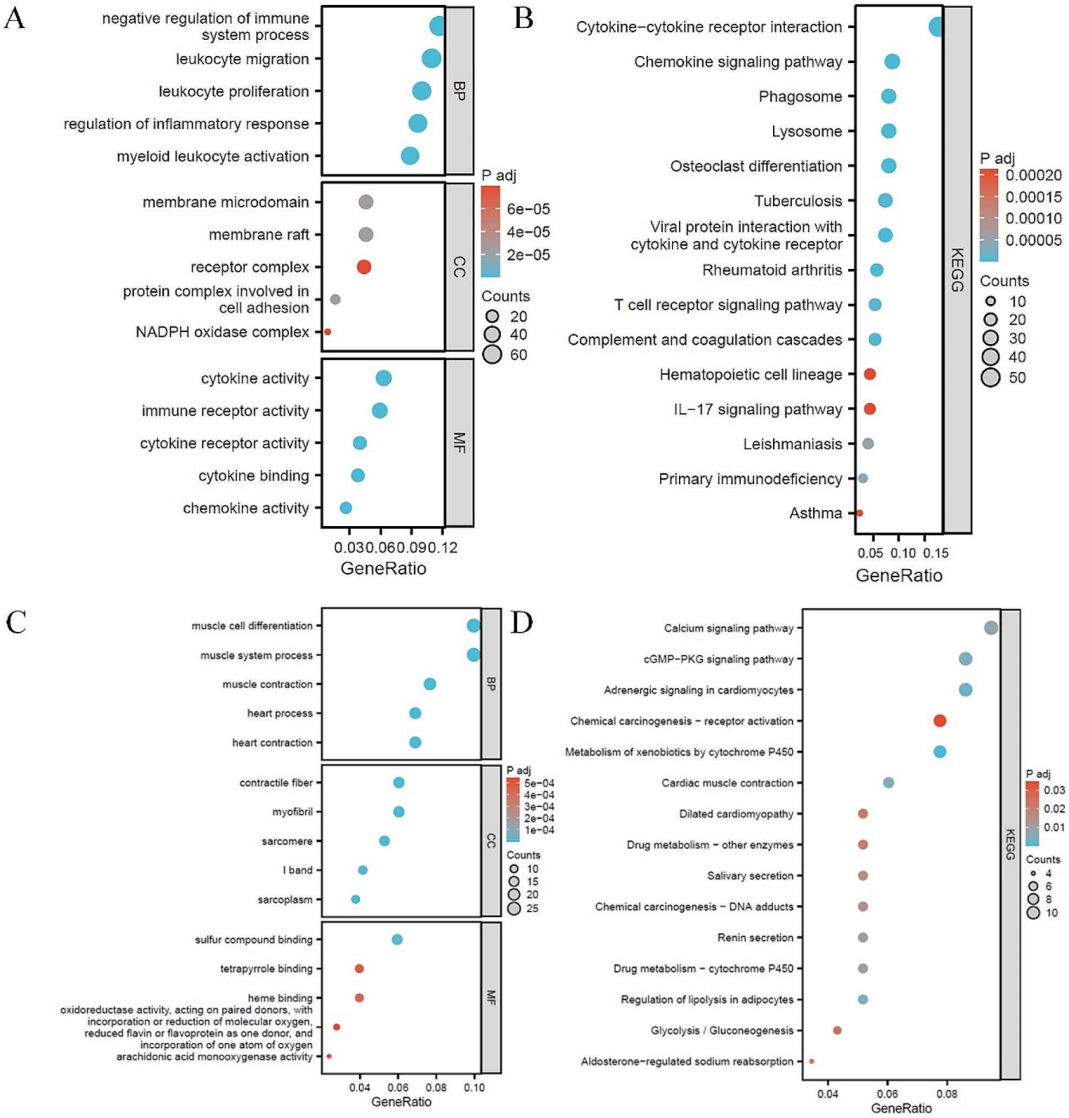
Functional analysis. (**A**). GO analysis of the key genes in the brown module; (**B**). KEGG analysis of the key genes in the brown module; (**C**). GO analysis of the key genes in the green module; (**D**). KEGG analysis of the key genes in the green module

Genes with SD > 1 (4999 genes) in the GSE49114 dataset was selected and subjected to WGCNA. Cluster analysis of the samples was performed and a heatmap was generated with clinical characteristics (Fig. 2A). According to the mean connectivity (Fig. 2B-C), a soft threshold of 18 (R^2^ = 0.87) was determined to construct co-expression networks, partition modules and merge similar modules (Fig. 2D). Based on the correlation analysis between groups and modules, two modules were identified to be highly correlated (cor > 0.8) with PAH: the brown and the green modules (Fig. 2E). Further module-to-module correlation analysis revealed a negative correlation between these two modules (Fig. 3A-B). In addition, GS vs. MM scatter plots (Fig. 3C-D) demonstrated significant positive correlations between the brown module and PAH (cor = 0.74, *p* = 1.1e-157), and also between the green module and PAH (cor = 0.71, *p* = 5.2e-79). Therefore, genes in these two modules were selected as key research objects for subsequent analysis. GS > 0.6 & MM > 0.8 were set as the criteria for screening key module genes, after which 612 key genes were screened out in the brown module and 294 in the green module. Finally, functional enrichment analysis was performed on these key genes within the green and brown modules (Fig. 4A-D), respectively. Corresponding bubble maps were plotted, showing GO and KEGG enrichment. Specifically, most of the key genes in the brown module were shown to be enriched in leukocyte migration, leukocyte proliferation, membrane microdomain, NADPH oxidase complex, cytokine activity, and cytokine-cytokine receptor interaction, etc. Meanwhile, key genes in the green module were revealed to be enriched in cGMP-PKG signaling pathway, contractile fibers, heme binding, and calcium signaling pathway, etc. These results suggest that these genes may influence the occurrence of PAH by regulating functions / pathways such as leukocyte, cytokine, and cGMP-PKG signaling pathway.

#### Single-cell analysis

**Fig. 5:**
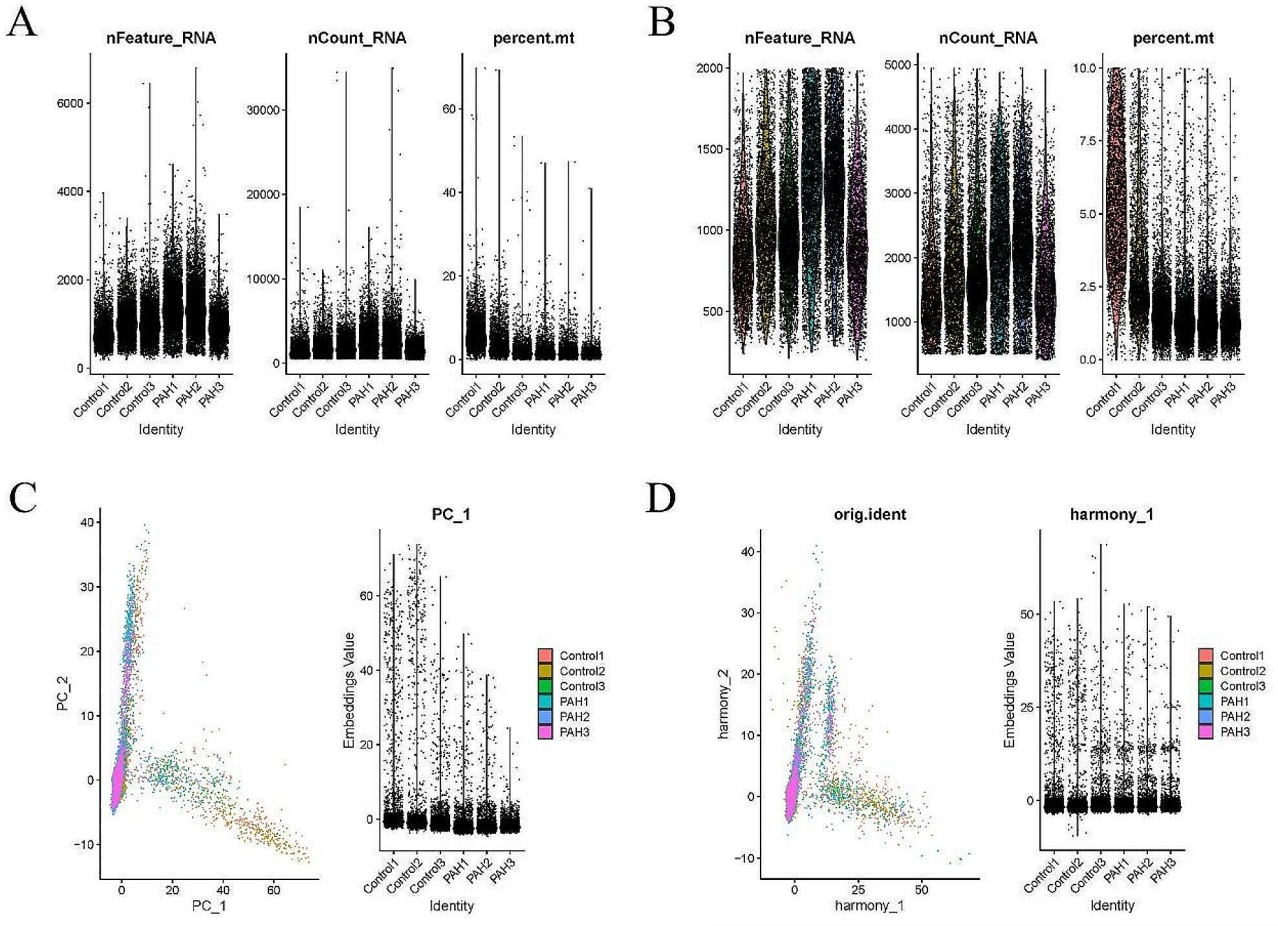
Data filtering and correction. (**A**). The violin plot shows the data before filtering; (**B**). The violin plot shows the data after filtering; (**C**). PC analysis and PC expression before data correction; (**D**). PC analysis and PC expression after data correction

**Fig. 6:**
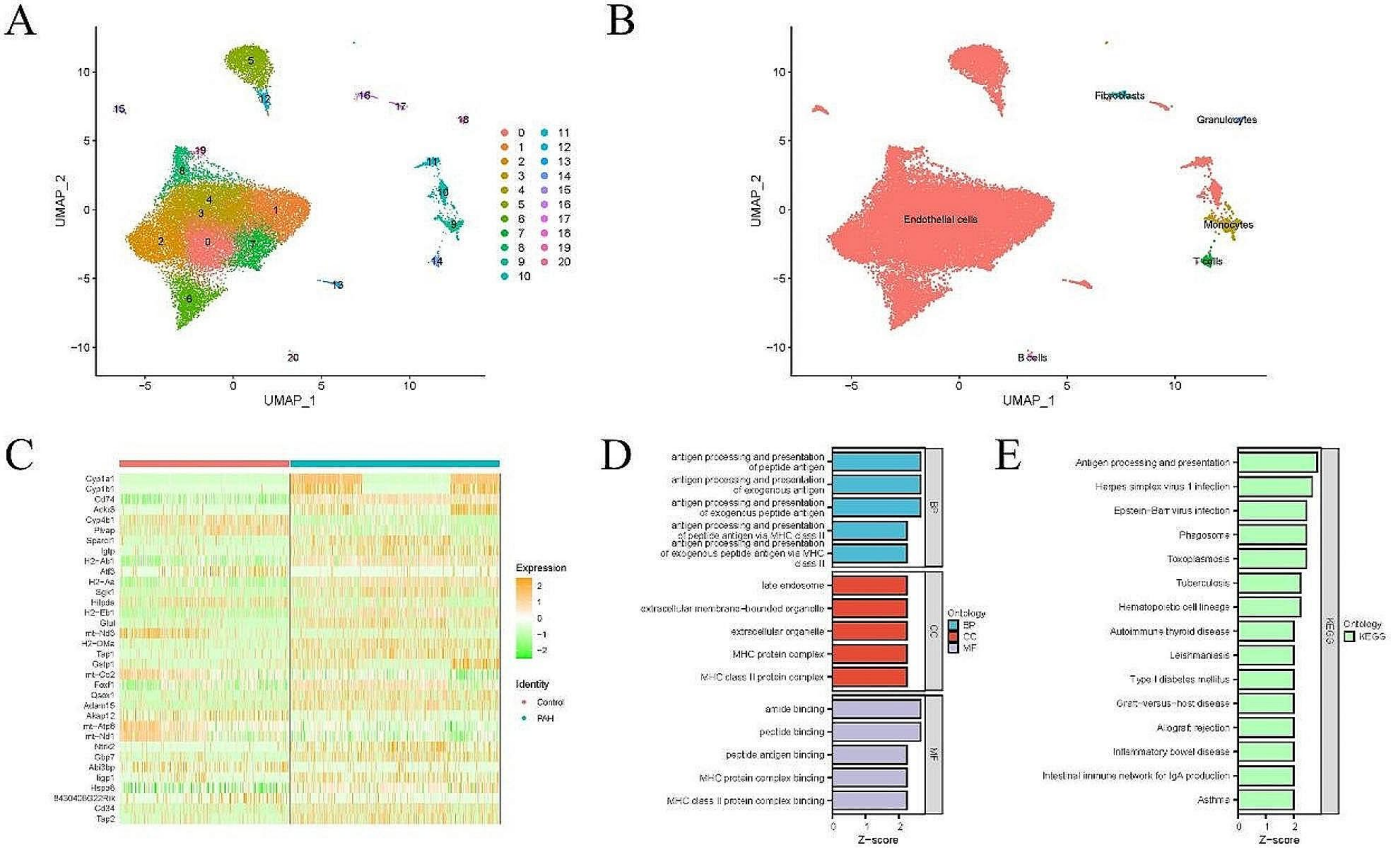
Single cell analysis. (**A**). Cell clustering analysis; (**B**). Cell annotation; (**C**). Heatmap of DEGs in ECs; (**D**). GO analysis; E. KEGG analysis

Single-cell analysis was performed on the GSE154959 dataset, and 200 < nFeature < 2000, percent. Mito < 10, and nCount < 5000 were set as the criteria for data filtering (Fig. 5A-B). Data were then batch-corrected to remove batch effects (Fig. 5C-D). The top 20 dim were selected for UMAP clustering analysis (Fig. 6A). Cell annotation based on these 20 dim of cells resulted in the identification of six types of cells: ECs, fibroblasts, granulocytes, monocytes, T cells, and B cells (Fig. 6B), with ECs accounting for the largest proportion. Subsequently, differential analysis of ECs was conducted between the two groups, and 34 DEGs were screened out based on |log_2_FC|>1 & pvalue < 0.05 as the standard (Fig. 6C). Finally, GO and KEGG analyses were performed on the DEGs, revealing that most of the genes were enriched in antigen processing and presentation of peptide antigen, late endosome and antigen processing and presentation, etc. (Fig. 6D-E).

### Screening of hub genes

**Fig. 7:**
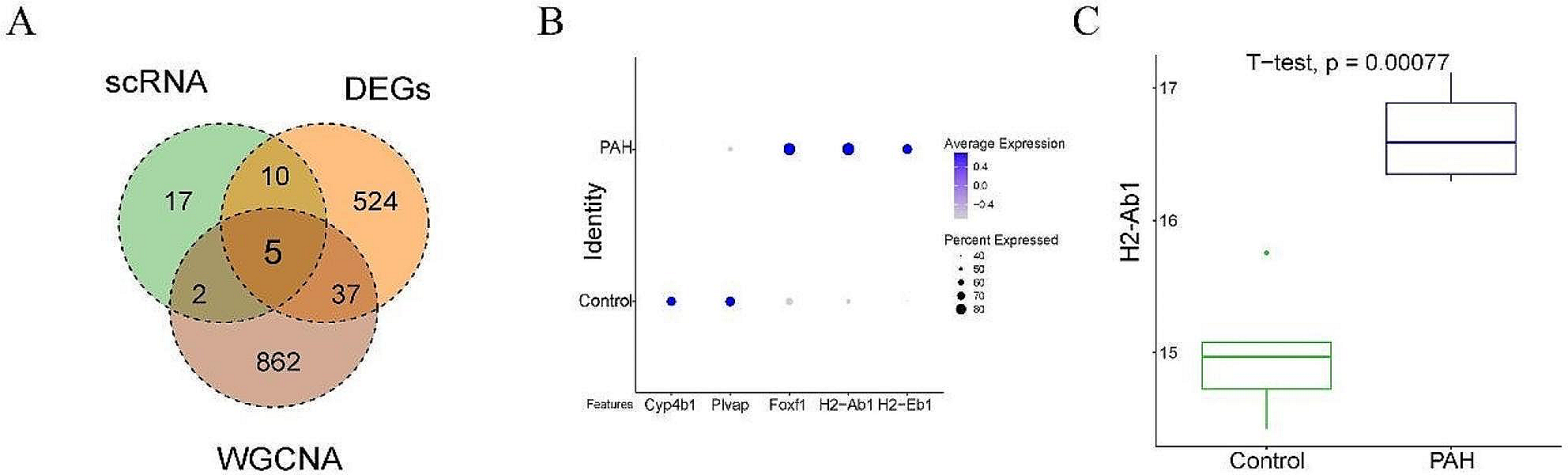
Screening of hub genes. (**A**). Venn diagram; (**B**). The expression of hub genes in the GSE154959 dataset; (**C**). Violin plot shows the expression of H2-Ab1 in the GSE180169 dataset

DEGs screened from the above three datasets were subjected to intersection, after which five key genes were screened out: Plvap, Cyp4b1, Foxf1, H2-Eb1, and H2-Ab1 (Fig. 7A). In the GSE154959 single-cell sequencing dataset, Plvap and Cyp4b1 were highly expressed, whereas Foxf1, H2-Eb1, and H2-Ab1 were lowly expressed in the control group (Fig. 7B). Moreover, analysis on the GSE180169 dataset indicated a downregulation of H2-Ab1 in the control group (Fig. 7C). Therefore, H2-Ab1 was selected for subsequent experiments.

#### H2-Ab1 was highly expressed in the lung of the SuHx mice

**Fig. 8:**
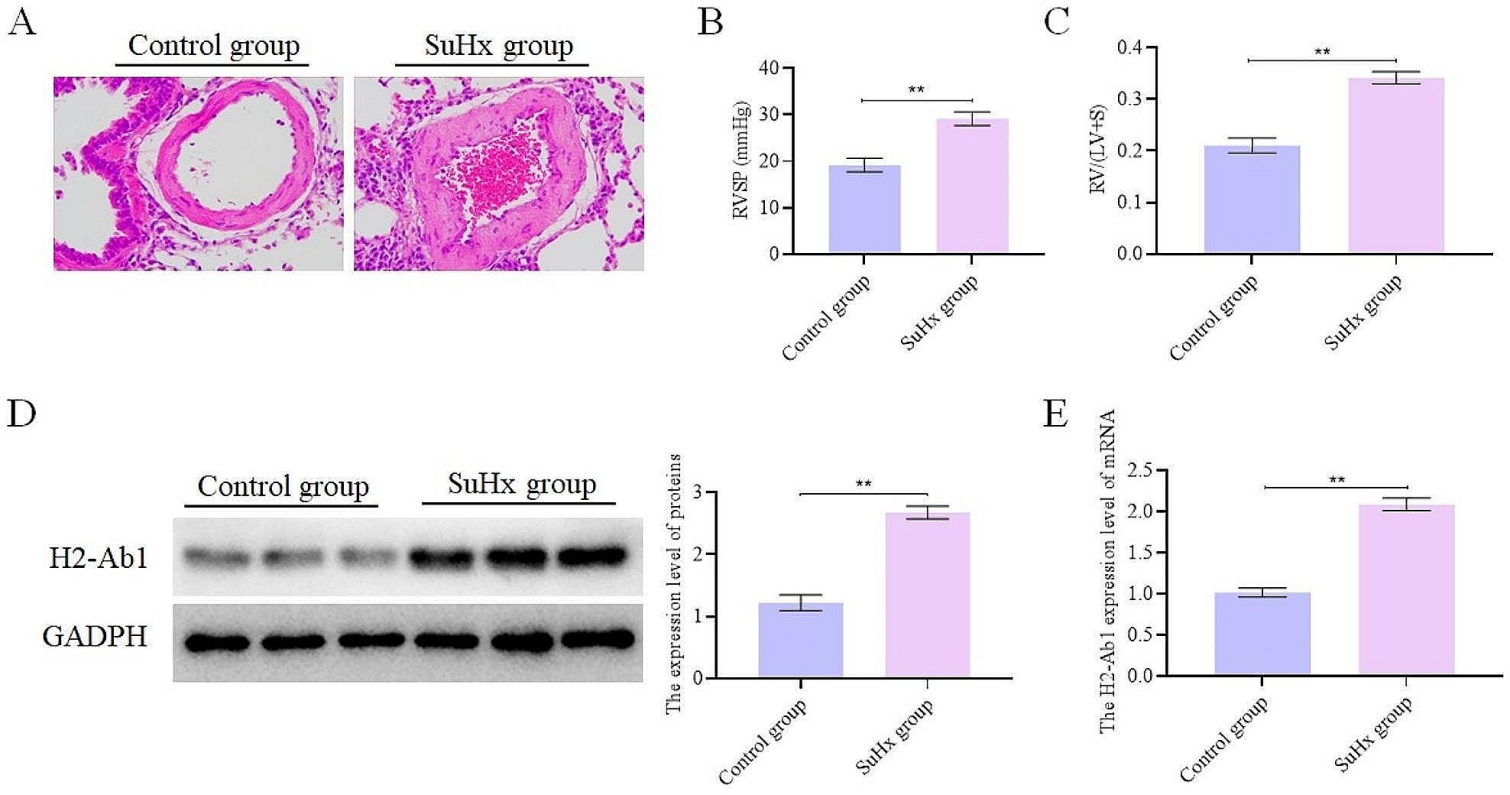
H2-Ab1 was highly expressed in the lung of the SuHx mice (*n* = 6). (**A**). HE staining results showing vascular remodeling; (**B**). RV systolic pressure in mice; (**C**). RV/ (LV + S) ratio; (**D**). WB results showing the expression of H2-Ab1 protein; (**E**). Detection of the expression of H2-Ab1. **p < 0.01

We selected H2-Ab1 for follow-up experiments after performing qRT-PCR detection on the five selected genes (Supplementary Fig. 1 and Supplementary Fig. 2) and carrying out relevant literature research. To validate the findings from bioinformatics analysis, the pathogenesis of PAH was simulated in mice using the SuHx method. Via HE staining, we observed vascular remodeling, vessel wall thickening, and inflammatory cell infiltration in pulmonary arterioles in the SuHx group, whereas these changes were absent in the control group (Fig. 8A). Furthermore, the RVSP and RV/ (LV + S) of the SuHx group were significantly increased (Fig. 8B-D and Supplementary Fig. 2). The above results proved the successful establishment of the PAH mouse model. Finally, the results of WB and qRT-PCR demonstrated a higher expression of H2-Ab1 in the SuHx group, significantly (Fig. 8E-F), which was also consistent with the findings from bioinformatics analysis.

#### H2-Ab1 was highly expressed in hypoxia-treated PAECs

**Fig. 9:**
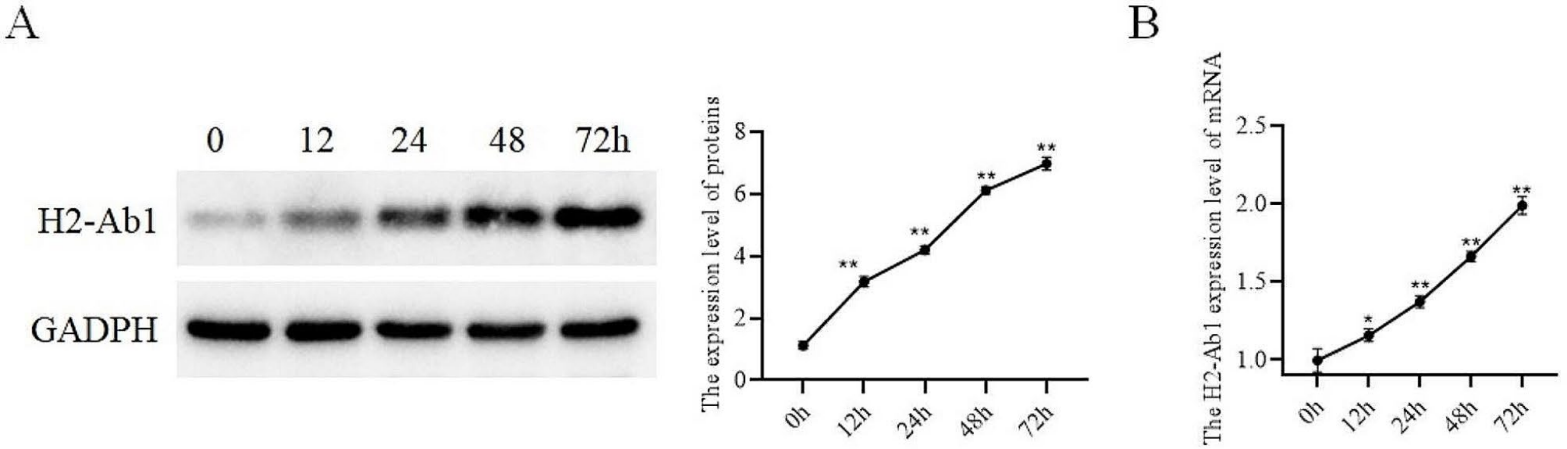
H2-Ab1 was highly expressed in hypoxia-treated PAECs. (**A**). Detection of the expression of H2-Ab1 in PAECs treated with hypoxia by WB; (**B**). Detection of the expression of H2-Ab1 in PAECs treated with hypoxia by qRT-PCR. *p < 0.05, **p < 0.01

We created the PAH cell model in vitro by treating PAECs with different durations (0, 12, 24, 48 and 72 h) of hypoxia. We used qRT-PCR and WB to detect the expression of H2-Ab1 in hypoxia-treated PAECs. The results consistently demonstrated a time-dependent increase in H2-Ab1 expression in hypoxia-treated PAECs (Fig. 9A-B and Supplementary Fig. 2), consistent with the findings from previous animal experiments.

#### H2-Ab1 could promote angiogenesis in hypoxia-treated PAECs

**Fig. 10:**
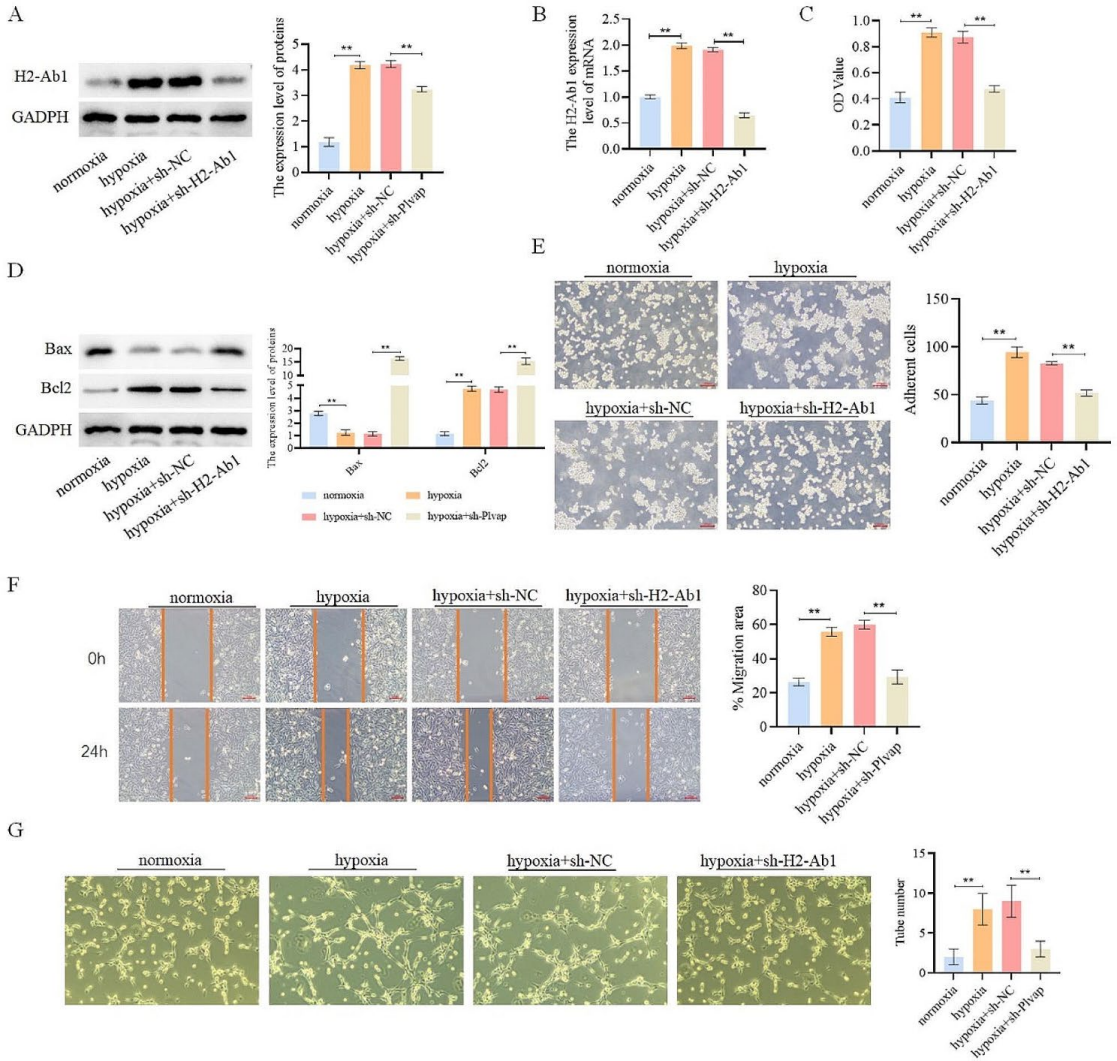
H2-Ab1 promoted angiogenesis in hypoxia-treated PAECs. (**A**). Detection of the expression of H2-Ab1 in each group of cells; (**B**). Detection of the expression of H2-Ab1 in each group of cells; (**C**). Detection of cell viability; (**D**). Detection of the expression of apoptosis-related proteins; (**E**). Detection of cell adhesion; (**F**). Detection of cell migration; (**G**). Detection of cell tubular formation ability through in vitro tubular formation experiment. **p < 0.01

H2-Ab1 is known for its high expression in hypoxia-treated PAECs. To investigate the impact of H2-Ab1 on the angiogenesis of PAECs, we downregulated H2-Ab1 expression in PAECs (Fig. 10A-B and Supplementary Fig. 2). The proliferation of PAECs was increased in the hypoxia group compared with the normoxia group, and decreased in the hypoxia + sh-H2-Ab1 group compared with the hypoxia + sh-NC group (Fig. 10C). We assessed apoptosis-related indicators (Bax and Bcl2) to evaluate cell apoptosis. The results demonstrated a decrease in the levels of apoptosis in the hypoxia group compared with the normoxia group, and an increase in the hypoxia + sh-H2-Ab1 group compared with the hypoxia + sh-NC group (Fig. 10D and Supplementary Fig. 2). Furthermore, we examined cell adhesion, migration and angiogenesis. By contrast with the normoxia group, the cell adhesion, migration, and angiogenesis were enhanced in the hypoxia group significantly. Conversely, when compared with the hypoxia + sh-NC group, the hypoxia + sh-H2-Ab1 group exhibited significantly decreased cell adhesion, migration, and angiogenesis (Fig. 10E-G). These results suggest that H2-Ab1 can regulate cell proliferation, apoptosis, adhesion, migration, and angiogenesis.

## Discussion

PAH, characterized by adverse arterial tree remodeling, leads to increased vascular resistance and subsequently elevated RV afterload [13]. To identify novel biomarkers for monitoring PAH progression, we retrieved PH-related datasets in the GEO database, and performed differential analysis, single-cell analysis, and WGCNA to screen genes related to PAH. Finally, five hub genes were screened by intersection: Plvap, Cyp4b1, Foxf1, H2-Ab1, and H2-Eb1. Functional analysis of the DEGs revealed their enrichment in items such as the Ras pathway, chemokine pathway, cytokine activity, and cGMP-PKG pathway. According to existing literature reports, mir-455-3p-1 can inhibit the expression of FGF7 by suppressing the RAS/ERK pathway, thereby inhibiting PAH [14]; Endothelial progenitor cell-derived exosomes were shown to inhibit the apoptosis and proliferation resistance of PASMCs in vitro by regulating the Ras-Raf-ERK1/2 pathway to affect the progression of PAH [15]; CXCR2, a chemokine receptor in ECs, was identified to exert a protective effect on PH by inhibiting vascular remodeling, and thus has a protective effect on PH [16]; macrophages can degrade IL-6 and prevent the occurrence of PAH [17]; Caspase-8 can activate macrophage-associated inflammation and produce IL-1β to promote PH [18]; The imbalance of NO-cGMP-PKG axis was shown to cause inflammation and thrombosis of pulmonary vessels, eventually leading to the occurrence of PAH [19]; cGMP-PKG emerged as a potential therapeutic target to restore reduced angiogenesis and mitochondrial biogenesis in persistent pulmonary hypertension of the newborn [20]. These functional analysis results collectively confirmed the correlation between the screened genes and PAH.

Plvap is a plasma membrane vesicle-associated protein, and is considered to be the main factor affecting endothelial cell permeability. It participates in the regulation of vascular permeability, and promotes angiogenesis and inflammation [21]. As reported, Plvap can promote angiogenesis in cholangiocarcinoma through the DKK1/CKAP4/PI3K pathway [22], and maintain the ultrastructure and function of chorionic capillaries in retinal ECs [23]. Moreover, Plvap plays a role in angiogenesis in liver cirrhosis [24], and is also considered to be a prognostic marker of glioma [25] and an early marker of glomerular endothelial injury in mice with diabetic kidney injury [26]. CYP4B1 can affect corneal inflammation and limbus vascular sprouting in rabbits [27]. In cancer, CYP4B1 demonstrates the capability to activate procarcinogens and facilitate angiogenesis [28]. In addition, CYP4B1 may hold significance in the prognosis of lung adenocarcinoma [29] and the progression of urothelial carcinoma [30]. Foxf1 is related to lung development [31], and extensive research has been conducted on this gene. FOXF1 has been confirmed to affect pulmonary angiogenesis in alveolar capillary dysplasia [32–34]. Both H2-Eb1 and H2-Ab1 are components the MHC class II protein complex and contribute to immune responses. However, there is limited research on the involvement of H2-Ab1, H2-Eb1 or MHC II in PAH. Relevant animal experiments have demonstrated that H2-Eb1 and H2-Ab1 affects the development of allergic rhinitis [35]; the expression of MHC II in the renal tubular affects renal fibrosis [36]; and MHC II plays crucial roles in tumor prognosis [37] and cancer metastasis [38]. Given the consistent expression trend of H2-Ab1 in both the GSE154959 and GSE180169 datasets, along with the findings from relevant literature reviews, we selected H2-Ab1 for subsequent experimental verification.

After successful modeling using the SuHx method in mice, H2-Ab1 exhibited a significantly increased expression in the model group, and showed a significant time-dependent up-regulation in hypoxia-treated PAECs. To further verify the role of H2-Ab1 in PAH, we down-regulated H2-Ab1 in PAECs and found that H2-Ab1 could regulate the proliferation, apoptosis, adhesion, migration, and angiogenesis of PAECs, thereby affecting the occurrence and development of PAH.

## Conclusions

In this study, we screened out PAH-related hub genes by bioinformatics methods, and selected H2-Ab1 for subsequent research based on a comprehensive literature review. At both the animal and cellular levels, H2-Ab1 expression was confirmed to be significantly increased in the constructed model. Additionally, at the cellular level, we confirmed that H2-Ab1 regulates the viability, apoptosis, adhesion, migration, and angiogenesis of PACEs. The findings of this study provide not only biomarkers for the early diagnosis and treatment of PAH, but also a scientific theoretical basis for the development of targeted drugs.

### Electronic supplementary material

Below is the link to the electronic supplementary material.

Supplementary Material 1

Supplementary Material 2

## Data Availability

The datasets generated and/or analysed during the current study are available in the GEO database (https://www.ncbi.nlm.nih.gov/geo/) (accession numbers: GSE49114, GSE180169 and GSE154959).

## Abbreviations

PAH: Pulmonary arterial hypertension
DEGs: Differentially expressed genes
HE: Hematoxylin-Eosin
WB: Western Blot
PAECs: Pulmonary artery endothelial cells
PH: Pulmonary hypertension
CTEPH: Chronic thromboembolic pulmonary hypertension
ECs: Endothelial cells
RV: Right ventricular
NO: Nitric oxide
PASMCs: Pulmonary artery smooth muscle cells
TPM: Transcript per million
SuHx: SU5416/hypoxia
LV + S: Left ventricular + septum
x ± s: Mean ± standard deviation
