# Supplementary figures and images for: Investigation into the role of H2-Ab1 in vascular remodeling in pulmonary arterial hypertension via Bioinformatics
Source: BMC Pulm Med. 2024 Jul 15;24:342. doi: 10.1186/s12890-024-03156-w (PMC11251127; doi:10.1186/s12890-024-03156-w)

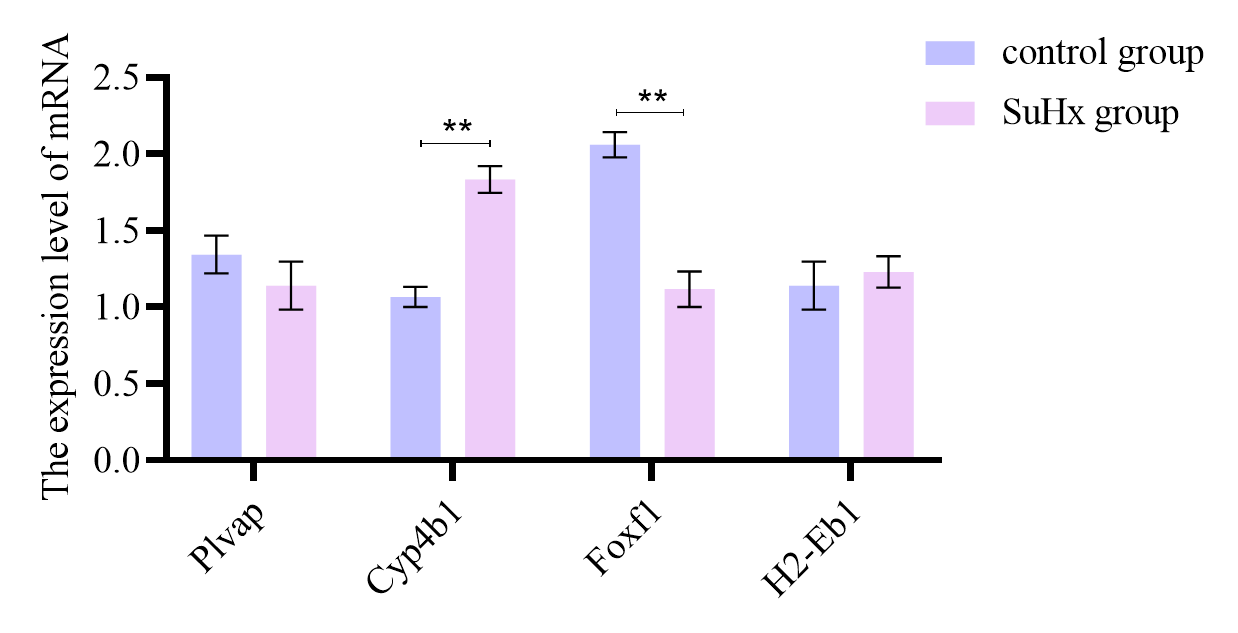

Supplement: Supplementary file 1 — Supplementary Material 1 [file 12890_2024_3156_MOESM1_ESM.tif]

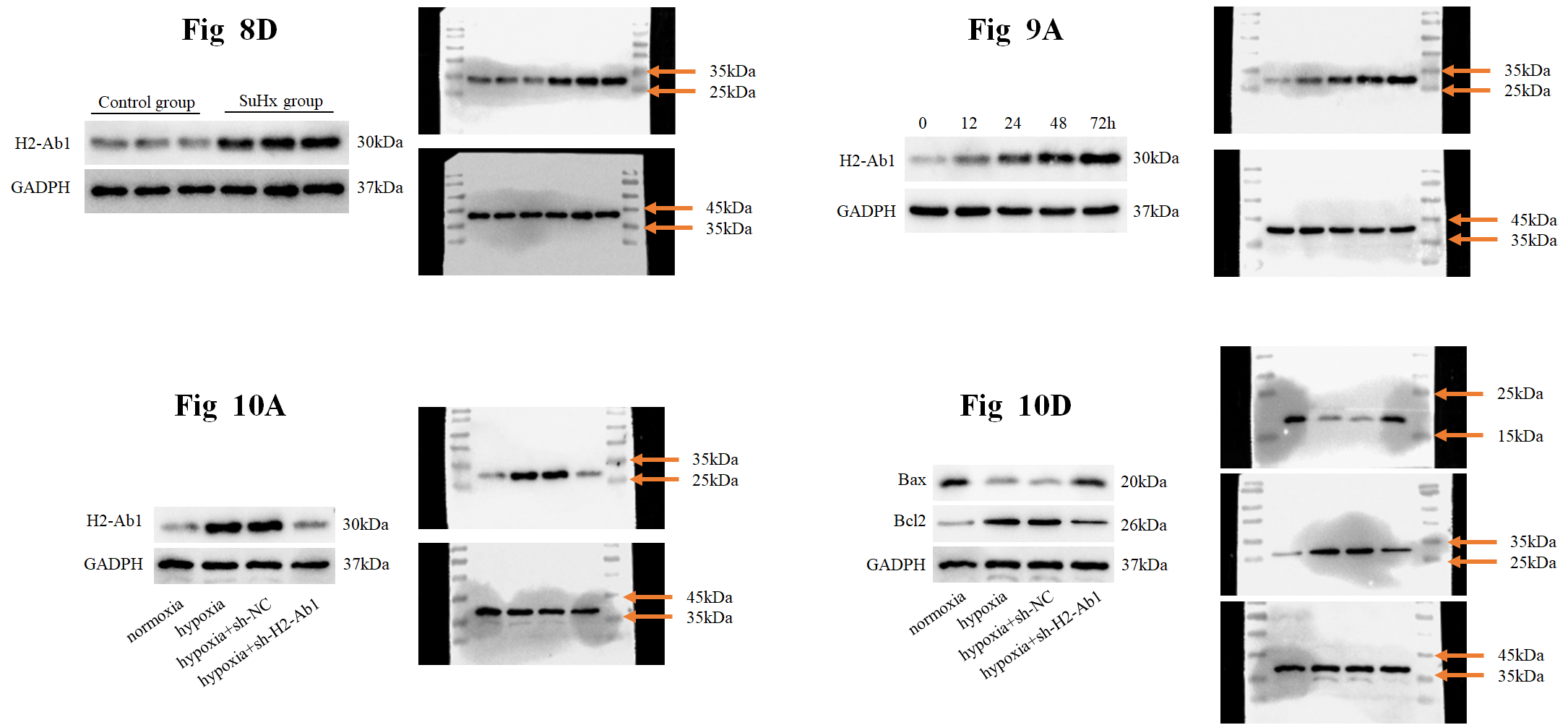

Supplement: Supplementary file 2 — Supplementary Material 2 [file 12890_2024_3156_MOESM2_ESM.tif]
